# The Treatment of Carbuncles

**Published:** 1910-01-29

**Authors:** 


### Surgery.

### THE TREATMENT OF CARBUNCLES.

The treatment of carbuncle in a young and vigorous adult is a matter which rarely gives rise to any anxiety. Free crucial incisions, removal of sloughs, and thorough swabbing with pure carbolic acid usually result in immediate cessation of the inflammatory process and speedy healing.

In the aged, the diabetic, or the subjects of chronic renal disease, the case is far different; and a carbuncle must be viewed with the gravest apprehension whatever its position or extent. Further, if its development is accompanied by but little pain, a low temperature, and a rapid pulse, then the prognosis is bad indeed. The active surgical interference which would be right and proper in a more normal individual is here contra-indicated. A free incision to give exit to pus is permissible.

But particular attention must be devoted to the general condition, and everything possible done to increase the resisting powers. A mistake that is too often made is to diminish the diet too suddenly; for although albuminuria or glycosuria must be treated on the general principles proper to the ailment, yet a too severe restriction of diet may prove the reverse of successful. To go carefully and gradually should therefore be the rule in attempting to reduce sugar or albumen.

In treating the actual carbuncle, a small incision under gas should be made, followed by the application twice daily of a cupping glass in which the pressure can be regulated by a rubber bulb; this passive congestion treatment will often succeed in staying the further progress of the lesion. In more extensive cases it is often physically impossible to apply Bier's treatment, owing either to the size or position of the carbuncle, although it is sometimes possible to get a glass over one or other of the openings with satisfactory results. Local treatment must, however, be assisted by measures calculated to increase the toxin-resisting powers of the body. In certain cases isolation of the organism (usually the staphylococcus aureus) and the preparation of a vaccine will be an important measure. But it must be remembered that in these cases the blood is not of such a quality that it can, even with the stimulus of a vaccine, produce an adequate amount of antitoxin, and the indication is therefore to provide the necessary antitoxin by the injection of antitoxic serum. A polyvalent anti-staphylococcal serum should be used in large quantities, the initial dose being 50-75 cubic centimetres. Further doses must be regulated by the amount of reaction and the progress of the local lesion.

The mode of its administration is of some importance. The patient is already infected with virulent organisms, his tissue resistance is low, and hypodermic injections of such a substance as serum in which organisms grow readily is especially liable in these cases to be followed by the development of an abscess. This danger is avoided by giving the serum per rectum. In order to avoid the possibility of the patient's unhappy state being rendered still more uncomfortable by the development of serum rash or joint pain, it is wise during the injections to administer calcium lactate in five-grain tabloids three times a day. The administration of horse serum alone is valuable, for there can be no doubt that it materially assists the antitoxic powers of the blood.

In regard to the local treatment of the carbuncle, 20 volume peroxide of hydrogen gently syringed into the wounds, acts as a powerful antiseptic, and is invaluable as a cleansing agent. The continued application of hot antiseptic compresses is to be deprecated, as it causes unpleasant irritation of the surrounding skin and is apt to lower its vitality. Wet sterile gauze is more satisfactory. An excellent dressing is the glycerine and clay paste, which has for a long time been in the U.S. Pharmacopœia. It has all the "comforting" properties of a linseed poultice, and is much more cleanly as well as being antiseptic. A single layer of gauze should be interposed between the skin and a thick layer of the warm clay poultice. At a later stage, when granulation is occurring, the rate of healing may be assisted by packing the wound with gauze soaked in tr. benzoin co. or lotio rubra. Needless to say, abundant fresh air, freedom from business cares and anxieties, good food of the right kind, and—if the patient is accustomed to alcohol—a little brandy, are adjuncts of the first importance in assisting a complete recovery.

